# Association between bowel habits and quality of bowel preparation for colonoscopy

**DOI:** 10.1097/MD.0000000000007319

**Published:** 2017-07-21

**Authors:** Dong-won Lee, Ja Seol Koo, Seonghee Kang, Seung Young Kim, Jong Jin Hyun, Sung Woo Jung, Hyung Joon Yim, Sang Woo Lee

**Affiliations:** Division of Gastroenterology and Hepatology, Department of Internal Medicine, Korea University College of Medicine, Seoul, Korea.

**Keywords:** bowel habits, colonoscopy, constipation, infrequent bowel movement, poor bowel preparation

## Abstract

The effectiveness of colonoscopy is highly dependent on the quality of bowel preparation. Although many studies have previously evaluated the role of cleansing methods and dosing regimens, few have examined the association between bowel habits and subsequent bowel preparation. Here, we aimed to evaluate the impact of bowel habits on the quality of bowel preparation.

A total of 404 patients who underwent a total colonoscopy and completed a personal bowel habit questionnaire at Korea University Hospital between December 2012 and December 2013 were enrolled. The usual stool form of patients was classified into 7 categories according to the Bristol Stool Scale (BSS). The quality of bowel preparation was determined during colonoscopy according to the Ottawa Bowel Preparation Scale (OBPS). Segment scores of ≥3 or total OBPS scores of >7 were defined as poor bowel preparation.

Poor bowel preparation was reported in 9.4% of observed colonoscopies. The odds ratio (OR) of poor bowel preparation being associated with infrequent bowel movements (<3/week) was 5.00 (95% confidence interval [CI], 1.91–13.1, *P* = .001). BSS types 1 and 2 tended to have an association with poor bowel preparation, but the association was statistically insignificant (OR: 2.38; 95% CI, 0.90–6.33, *P* = .082). After adjusting for age, sex, drinking, presence of diabetes mellitus, and bowel preparation regimen, infrequent bowel movement (<3/week) was still significantly associated with poor bowel preparation. When subdividing by colonic segment, it was significantly associated with poor bowel preparation in all segments.

Infrequent bowel movement (<3/week) was significantly associated with poor bowel preparation.

## 1 Introduction

Colonoscopy is a useful tool for the prevention of colorectal cancer.^[1,2]^ The effectiveness of colonoscopy is largely dependent on the quality of bowel preparation, and poor preparation is associated with a low detection rate of colonic polyps and prolonged procedure time, including cecal intubation and withdrawal time.^[3–5]^ Previous studies on bowel preparation have focused on the roles of cleansing methods and dosing regimens, and little is known regarding the association between bowel habits and bowel preparation.^[6–8]^ Constipation has been reported as a probable risk factor for poor bowel preparation in several studies; hence, infrequent bowel movements are thought to be associated with poor bowel preparation.^[9,10]^ However, because the definition of constipation is somewhat subjective and includes several heterogeneous factors, such as excessive straining, hard stool, sense of incomplete bowel evacuation, and infrequent bowel movements, the exact relationship between bowel habits and the quality of bowel preparation remains unclear. Additionally, most studies were limited in that constipation was either recorded as a yes/no response of the patient or defined arbitrarily by the number of bowel movements per week.^[10,11]^

According to previous studies, bowel habits (bowel movement frequency and stool consistency) reflect whole or colonic transit time, and they are simply measured at outpatient clinics based on taking patient's medical history.^[12]^ Therefore, it is of great value to evaluate the impact of bowel habits on the quality of bowel preparation for colonoscopy, but data concerning this issue are very limited.

In the present study, we aimed to better characterize the relationship between personal bowel habits and quality of bowel preparation, and to investigate the risk factors associated with poor bowel preparation.

## 2 Methods

### 2.1 Subjects

This retrospective study included all patients who underwent total colonoscopy for the purpose of screening or diagnostic evaluation and completed a bowel habit questionnaire at the gastroenterology outpatient clinic of the Korea University Hospital between December 2012 and December 2013. A total of 404 outpatients were analyzed in the study after excluding patients who did not complete the bowel preparation regimen or did not provide an adequate response on the bowel habit questionnaire, patients with a history of colorectal surgery, patients less than 20 years or more than 80 years old, and motor impaired patients with a performance status 3 or 4 due to underlying comorbidities such as strokes. We also excluded patients taking drugs with substantial effects on gastrointestinal motility including prokinetics, stool softeners (e.g. magnesium), secretomotor stimulants (e.g., bisacodyl), or anticholinergics for non-gastrointestinal purposes (e.g., antidepressants, antihistamines).

Clinical and demographic data were extracted from each patient's baseline questionnaire and included age, sex, level of education, smoking status, alcohol consumption, previous history of colonoscopies, and medical history of chronic disease (such as hypertension and diabetes mellitus [DM]). This study was approved by the institutional review board of Korea University Ansan Hospital. The need for informed consent was waived in view of the retrospective observational design of the study.

### 2.2 Bowel habits assessment

Bowel movement frequency (the number of bowel movements) was ascertained by asking subjects the closed-ended question “How often in a week do you usually have a bowel movement?” Based on the Rome III diagnostic criteria of functional constipation (<3/week) and the World Health Organization definition of diarrhea (>2/day), answers were classified into the following 3 different categories: <3/week (infrequent), 3 to 14/week (normal), >14/week (frequent).

Information regarding stool consistency was acquired and classified into 7 different categories in accordance with the Bristol Stool Scale (BSS); the categories are easily expressed as an image on the questionnaire itself.^[13,14]^ BSS categories were further classified into 3 main categories as follows: hard form: BSS type 1 or 2; normal form: BSS type 3, 4, or 5; and (3) loose form: BSS type 6 or 7.

### 2.3 Quality of bowel preparation

Prior to the scheduled colonoscopy, study subjects were advised to avoid solid food for 1 day and fast for at least 12 hours. Four liters of polyethylene glycol (PEG) solution or 1 L clear liquid with 2 L of PEG with ascorbic acid was used as a bowel purgative. In cases where 4 L of PEG was used, 2 L of PEG was administered the night before the colonoscopy and the remainder was administered on the morning of the procedure. Colonoscopies were performed within 4 to 7 hours after consuming the second dose of the preparation agent in the morning. The procedures were performed by experienced attending endoscopists by using an Olympus CF 260 colonoscope (Olympus Medical Systems, Tokyo, Japan). Midazolam and propofol were used for sedation. After completing the colonoscopy examination, the endoscopists rated the quality of the preparation by using the established and validated Ottawa Bowel Preparation Scale (OBPS).^[15]^

In the OBPS, bowel cleanliness and fluid volume are assessed separately. Each major segment of the colon (the right, mid, and rectosigmoid [RS] colon) was individually assessed for bowel cleanliness and was rated using a scale from 0 to 4. In each segment, a score of 3 or greater was indicative of a poor bowel preparation, as residual stool prevented clear observation of the mucosa despite washing and suctioning. In addition to the segment score, the fluid score was reflective of fluid quantity throughout the colon and was rated using a scale from 0 to 2. A score of 0 indicated a small amount of fluid, whereas a score of 1 indicated a moderate amount and 2 indicated a large amount. In contrast to bowel cleanliness, fluid amount per se was not regarded as a major determinant of a poor bowel preparation because the effect of fluid amount on the quality of bowel preparation largely depended on bowel cleanliness. However, for reflecting the indirect effect of fluid amount on bowel preparation, the total colon preparation score was calculated by adding each of the 3 major segment scores to the fluid score. It ranged from 0 to 14, and a score of 8 or greater was indicative of poor bowel preparation. In summary, segment scores of ≥3 or total OBPS scores of >7 were defined as poor bowel preparation in this study.

### 2.4 Statistical analysis

The value of each continuous variable is expressed as the mean ± standard deviation. Each categorical or discrete variable is presented as a percentage. Differences between the 2 groups were analyzed using the chi-squared or Student's *t* test. Logistic regression was used to calculate the odds ratio (ORs) with 95% confidence intervals (95% CI) for evaluating the risk for poor bowel preparation. Covariates used in our multivariate analyses included variables with a significant result on the univariate test (*P* < .100) in addition to those factors associated with poor bowel preparation from a previous study (such as sex, age, and bowel preparation regimen). Statistical analyses were performed using SPSS (version 18.0 for Windows, Chicago, IL). All tests were 2-tailed, and a *P* value of <.05 was considered statistically significant.

## 3 Results

All 404 patients who underwent a total colonoscopy and completed the bowel habit questionnaire were included in this analysis. The mean age of patients was 53.2 ± 12.8 years and 218 (54.0%) patients were men. Poor bowel preparation was reported in 38 (9.4%) of the observed colonoscopies. When analyzed by colon segment, 28 were in the right colon (6.9%), 7 were in the mid colon (1.7%), and 9 were in the RS colon (2.2%). Eleven patients had 2 or more colon segments with poor bowel preparation; among these, 2 patients had poor bowel preparation in the right and mid colon, 3 in the right and RS, and 4 in all segments.

Baseline patient characteristics and their distribution across the adequate and poor bowel preparation groups are summarized in Table 1. The baseline parameters of age, sex, smoking and drinking status, indication for colonoscopy, education level, and bowel preparation regimens did not differ between the 2 groups. However, patients with DM or infrequent bowel movements (<3/week) were more prevalent in the poor bowel preparation group (both *P* = .003). Although the distribution of stool consistency type was not significantly different between the adequate and poor bowel preparation groups, the latter tended to have more than double the percentage of types 1 and 2.

**Table 1:**
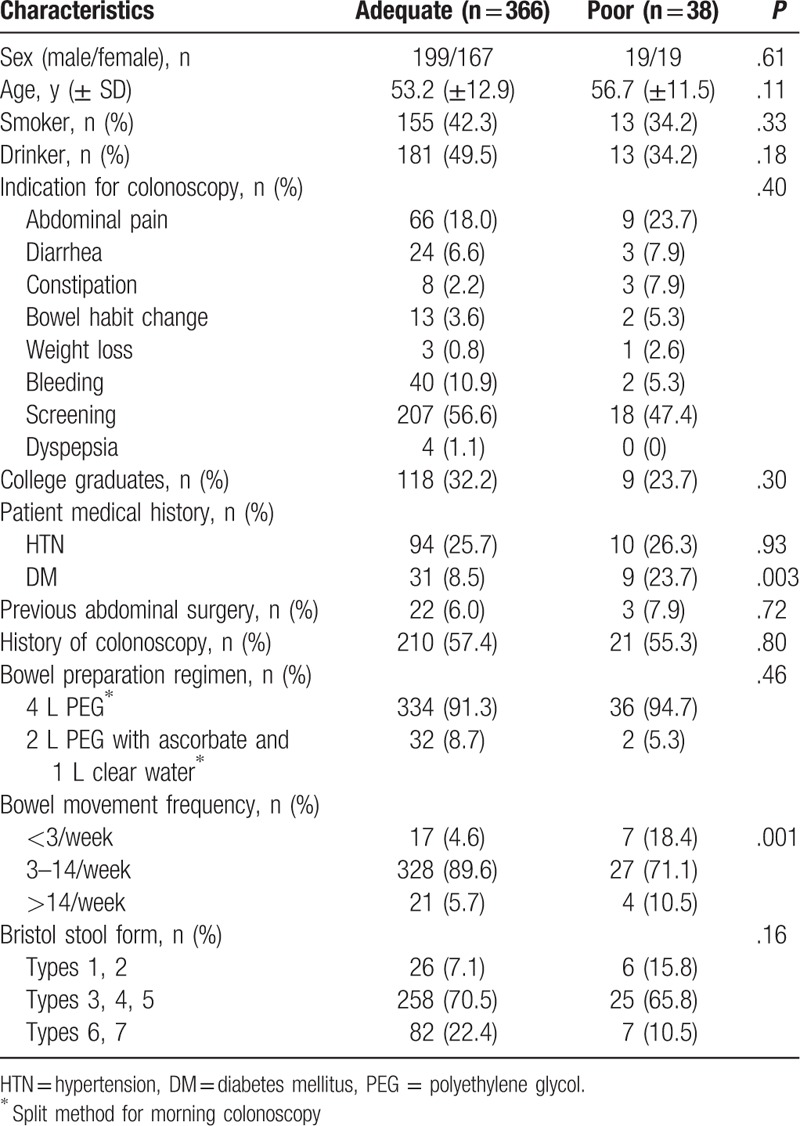
Baseline characteristics of the patients.

**Figure 1:**
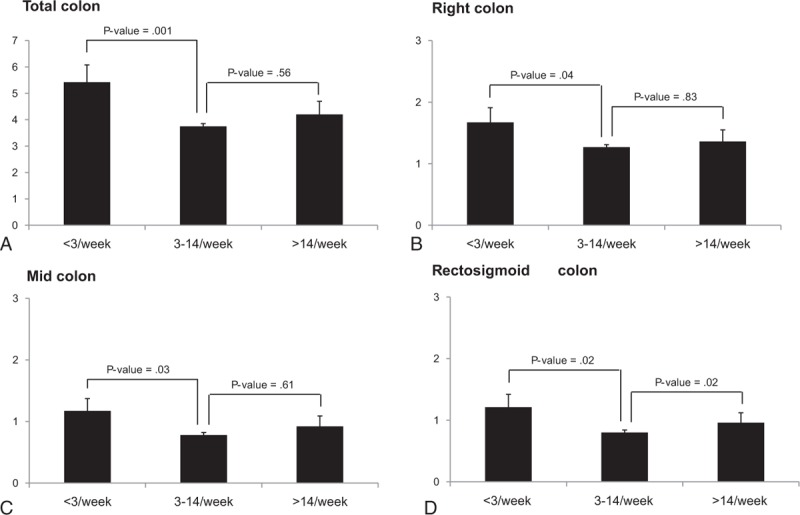
Ottawa Bowel Preparation Scale (OBPS) score for total colon and each colonic segment according to bowel frequency (A) total colon, (B) right colon, (C) mid colon, (D) rectosigmoid colon.

Figure 1 shows the average OBPS score for the total colon and each colonic segment according to bowel movement frequency. The OBPS score of the infrequent bowel movements group was significantly higher than that of the normal bowel movements group in total and in all colonic segments (5.42 ± 3.21 vs 3.75 ± 1.97 in the total colon, 1.67 ± 1.17 vs 1.27 ± 0.73 in the right colon, 1.17 ± 0.96 vs 0.78 ± 0.70 in the mid colon, 1.21 ± 1.02 vs 0.80 ± 0.04 in the RS colon). However, there was no difference between the normal and frequent bowel frequency groups.

Possible clinical factors related to poor bowel preparation and their calculated ORs are presented in Table 2. DM and bowel movement frequency were significantly associated with poor bowel preparation in the univariate analysis. Infrequent bowel movement was associated with an increased risk of approximately 5-fold, compared to that associated with normal bowel movements (OR, 5.00; 95% CI, 1.91–13.1). The OR of BSS types 1 and 2 being associated with poor preparation was 2.38 (95% CI, 0.90–6.33), but it was statistically insignificant. Multivariate analysis adjusted for age, sex, drinking, presence of DM, and bowel preparation regimen showed that less than 3 bowel movements per week was an independent risk factor for poor bowel preparation (OR, 5.20; 95% CI, 1.79–15.2).

**Table 2:**
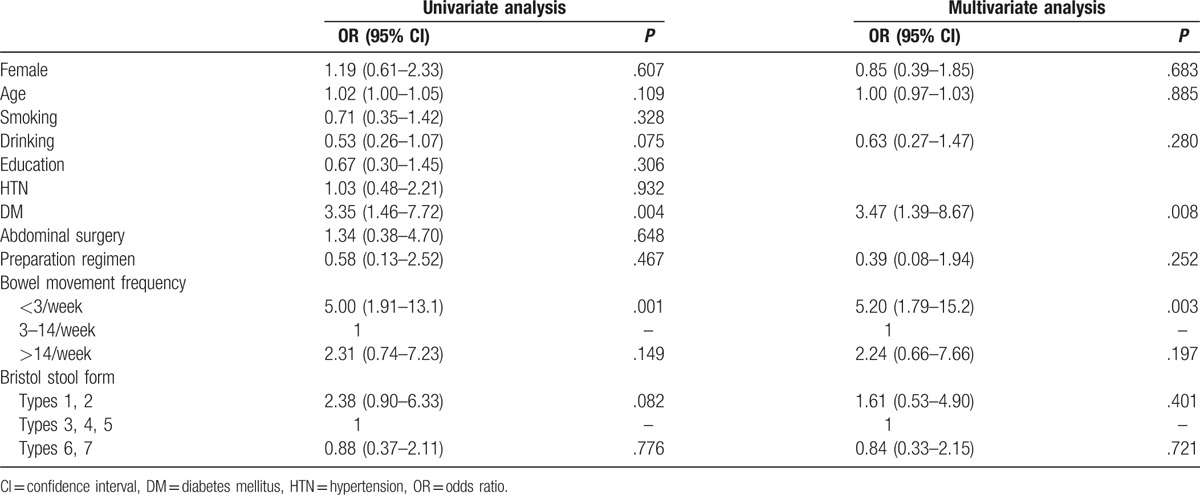
Univariate and multivariate logistic regression analysis for clinical factors of poor bowel preparation.

Table 3 shows clinical factors associated with poor bowel preparation in each colonic segment. Infrequent bowel movements were associated with poor bowel preparation in all colonic segments (OR, 6.08; 95% CI, 1.86–19.8 in the right colon; OR, 9.30; 95% CI, 1.21–71.6 in the mid colon; OR, 24.8; 95% CI, 4.03–153.0 in the RS colon).

**Table 3:**
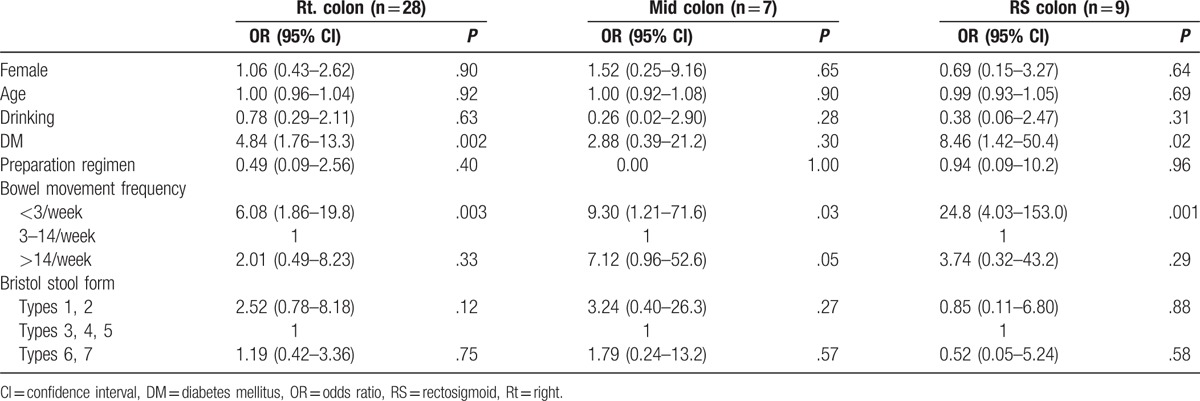
Clinical factors associated with poor bowel preparation in each colon segment.

Interestingly, DM was also significantly associated with poor bowel preparation in both univariate and multivariate analyses.

## 4 Discussion

The results of this study provide evidence of an association between bowel habits and poor bowel preparation. Infrequent bowel movements (<3/week) increased the risk of poor bowel preparation by about 5-fold. Furthermore, DM was shown be an independent risk factor for poor bowel preparation.

Predictors of bowel preparation have been previously identified in several studies and include age, male sex, comorbidities such as DM and stroke, previous abdominal surgery, and poor socioeconomic status.^[9,16,17]^ However, in the present study, patient-related factors (with the exception of infrequent bowel movements and DM) were not significantly associated with poor bowel preparation.

Previous studies have described a relationship between a self-reported history of constipation and inadequate bowel preparation.^[9,18]^ However, these studies were limited in that constipation was either recorded as a yes/no response by the patient or was defined arbitrarily by the number of bowel movements per week. Because the definition of constipation was heterogeneous and the normal range of bowel movements was undecided, the exact relationship between the bowel habits of constipated patients and bowel preparation is not clearly defined. The present study was more comprehensive in that it used 2 clinical features of bowel habits—that is, the number of bowel movements per week and stool consistency graded by the BSS scale. These were easy to understand by the respondents and easy to quantify, thus making it possible to correlate each feature independently with an endoscopic assessment of bowel preparation. Bloom et al^[18]^ reported that as the number of daily reported bowel movement increases, the Ottawa score decreases independent of the preparation type, which is consistent with the results of the present study.

In this study, we found that the frequency of poor bowel preparation in the right colon was about 2 times higher than that in other segments. The underlying mechanism is not clear in the present study. The speed of digestive fluid moving through the small intestine decreases abruptly as soon as it enters the colon, which might make it easy for residue to be deposited in the right colon. Moreover, the direction of flow is against gravity in the right colon; hence, any residue such as food or fecal material may not be easily flushed out by the preparation fluid.

Hard stool consistency is an important factor in the ROME III diagnostic criteria for constipation and tends to be associated with poor bowel preparation.^[12]^ However, in the present study, stool consistency measured by BSS was not an independent predictor of poor bowel preparation, although hard stool was more prevalent in the poor bowel preparation group. This might be because of the small sample size and inaccurate self-reporting of BSS from respondents, which was inevitable in our study design. Several studies, including that of Saad et al, have shown that stool form evaluated by BSS is moderately correlated with either whole gut or colonic transit times.^[13,19,20]^ However, as the majority of these studies had small sample sizes and significant methodological limitations, future well-designed large-scale studies are needed to evaluate the effect of stool consistency on bowel preparation.

In the present study, DM was associated with poor bowel preparation. Previous studies have reported that DM patients have slower colonic transit and longer evacuation times when compared with non-DM patients, which support our results.^[21,22]^

This study had several limitations. First, as clinical information from subjects including bowel habits was obtained from a questionnaire, response bias is expected. In addition, other factors known to affect bowel movement and stool formation, including current medication, were not fully evaluated. However, as this study directly recorded the patient's recent bowel habits and the colonoscopy was performed close to the time of questionnaire completion, the association of bowel habits with the quality of bowel preparation may be more easily clarified.

In summary, infrequent bowel movements (<3/week) were significantly associated with poor bowel preparation. It is critical for clinicians to be able to identify predictors for the quality of bowel preparation based on a patient's medical history. On the basis of our results, fewer than 3 bowel movements per week might be a useful indicator to guide bowel preparation. We further suggest that management of diet and additional medications might be necessary in high-risk patients, as classified by bowel movement frequency.
